# Enhancing optoacoustic mesoscopy through calibration-based iterative reconstruction

**DOI:** 10.1016/j.pacs.2022.100405

**Published:** 2022-09-23

**Authors:** Urs A.T. Hofmann, Weiye Li, Xosé Luís Deán-Ben, Pavel Subochev, Héctor Estrada, Daniel Razansky

**Affiliations:** a Institute for Biomedical Engineering and Institute of Pharmacology and Toxicology, Faculty of Medicine, University of Zurich, Switzerland; b Institute for Biomedical Engineering, Department of Information Technology and Electrical Engineering, ETH Zurich, Switzerland; c Institute of Applied Physics, Russian Academy of Sciences, Nizhny Novgorod, Russia

**Keywords:** Optoacoustic mesoscopy, Photoacoustic imaging, Quantitative reconstruction, Iterative inversion, Impulse response, Biomedical imaging, Skin imaging

## Abstract

Optoacoustic mesoscopy combines rich optical absorption contrast with high spatial resolution at tissue depths beyond reach for microscopic techniques employing focused light excitation. The mesoscopic imaging performance is commonly hindered by the use of inaccurate delay-and-sum reconstruction approaches and idealized modeling assumptions. In principle, image reconstruction performance could be enhanced by simulating the optoacoustic signal generation, propagation, and detection path. However, for most realistic experimental scenarios, the underlying total impulse response (TIR) cannot be accurately modelled. Here we propose to capture the TIR by scanning of a sub-resolution sized absorber. Significant improvement of spatial resolution and depth uniformity is demonstrated over 3 mm range, outperforming delay-and-sum and model-based reconstruction implementations. Reconstruction performance is validated by imaging subcutaneous murine vasculature and human skin in vivo. The proposed experimental calibration and reconstruction paradigm facilitates quantitative inversions while averting complex physics-based simulations. It can readily be applied to other imaging modalities employing TIR-based reconstructions.

## 1 Introduction

Optoacoustic mesoscopy (OAM) resolves blood vessels and other light-absorbing structures beyond the depth limits imposed by strong light scattering in biological tissues [1], [2], [3]. The method is based on scanning a spherically focused single-element ultrasound (US) detector across a biological sample diffusely illuminated with nanosecond-duration laser pulses. Absorbers within the optical path of light convert photon energies into heat, which results in thermoelastic expansion. The local deformation leads to an initial pressure rise followed by the emission of high frequency US waves detected with the spherical sensor located outside the imaged specimen [4], [5].

The unique properties of OAM have enabled optical imaging of living tissues at previously unreachable resolution-depth range, which fostered the development of more advanced hardware implementations. Of particular importance is the development of highly sensitive and broadband US detectors based on polyvinylidene fluoride (PVDF) films. PVDF sensors accurately detect broadband low-intensity OAM signals with improved signal-to-noise ratio (SNR) [6]. Light delivery through a central aperture of custom-made detectors further contributed to an enhanced sensitivity and uniformity of the illumination beam across the detector sensitivity field [7], [8]. On the other hand, acceleration of mechanical scans enabled fast mesoscopic imaging of large field-of-views (FOVs) while reducing motion artifacts [9], [10], which facilitated new biological and medical applications. Customized trigger schemes and optical setups further allowed for simultaneous acquisition of multiple wavelengths for functional imaging in a single mechanical scan [10].

A typical OAM image can simply be formed by superimposing the detected waveforms (A scans) acquired with the US detector at every position of the scanned image grid. However, image reconstruction algorithms could effectively combine the information from the neighbouring time domain signals to enhance the spatial resolution, SNR, and contrast-to-noise ratio (CNR) of the images, particularly for absorbers positioned outside of the focal plane of the spherically focused US detector. One example is the synthetic aperture focusing technique (SAFT), which was adapted from pulse echo US imaging or other coherent imaging modalities [11], [12], [13]. In OAM, the SAFT approach essentially reduces the focused detector shape into a virtual point receiver positioned at the focal point. Reconstruction is then performed by summing the contributions of neighbouring signals on equal-delay arcs surrounding this point. Coherence factor weighting in combination with SAFT has been shown to further improve the resolution and reduce noise in the reconstructed images for point like absorbers [14], [15]. Sensitivity field weighting was shown to also compensate for depth-dependent intensity differences during reconstruction [16]. Other approaches include deconvolution methods based on a theoretically calculated point spread function (PSF) after SAFT providing enhanced lateral and axial resolution beyond the acoustic diffraction limit [10], [17], [18] and implementations in the Fourier domain improving reconstruction speed and depth uniformity [19].

A more accurate alternative for acoustic inversion is provided by the model-based iterative reconstruction (MBR) algorithms. In this case, the total impulse response (TIR) is commonly taken into account by simulating the known optoacoustic excitation and detection path. The modeled TIRs can additionally account for acoustic propagation effects as well as the spatial impulse response (SIR) of the transducer array [20]. The incorporation of optical models has been demonstrated to increase the effective imaging depth [21]. In a similar fashion, advanced MBR algorithms based on a simulated TIR (sTIR) have been shown to significantly enhance imaging performance in optoacoustic tomography systems [22], [23], [24], [25], [26]. Recently, we have also shown that MBR based on a sTIR can also enhance OAM images. Specifically, an algorithm relying on the translational symmetry of the transducer sensitivity field along the horizontal directions was shown to improve the spatial resolution, SNR, and CNR of the reconstructed optical absorbance distribution beyond the capabilities of SAFT [27].

**Fig. 1:**
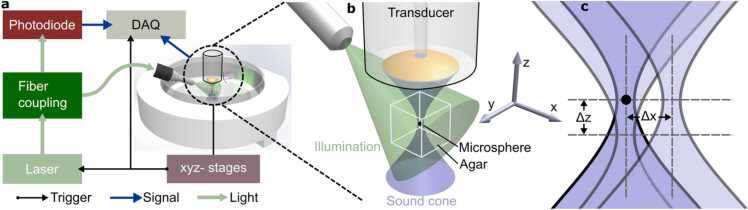
Schematic representation of the proposed experimental calibration procedure. a) A microsphere is moved to different positions in the sensitivity field of the transducer using motorized stages. At each stage position laser and data acquisition are triggered using customized electronics. b) To decouple the acoustic and optical propagation problems, illumination is provided from the side while scanning over the microsphere which is embedded in an agar block to avoid motion artifacts. c) The 4D TIR is then sampled over a three-dimensional region covering 1 mm in the lateral direction and 3 mm in the axial direction around the focal point.

In this work, we performed an experiment to extract the calibrated TIR (cTIR) required for accurate MBR (Fig. 1). For this, a sub-resolution microsphere was scanned volumetrically around the focal point of the PVDF transducer. The measurement accurately accounts for the electrical impulse response (EIR) of the transducer and the amplification electronics as well as imperfections in the sensitivity field that cannot be accurately accounted for when using the theoretical simulation approach. The MBR was accelerated using modern graphics processing units (GPUs). We showcase the quantitative improvements provided with the proposed cTIR-based MBR (cMBR) against SAFT and sTIR-based MBR (sMBR) in phantom measurements. Finally, subcutaneous murine vasculature and human skin were imaged to demonstrate the in vivo imaging potential of the developed approach.

## 2 Methods

### 2.1 Optoacoustic mesoscope

Three lasers (Onda 532 nm, Bright solutions, Italy; Onda 1064 nm, Bright solutions, Italy; Credo Dye laser, Sirah Lasertechnik, Germany) were combined in a single optical path for the optoacoustic signal excitation [10]. 1% of the combined beam was redirected into a fast, pre-charged photodiode (PD; DET10A2, Thorlabs, USA), while 99% of the energy was coupled into a multimode fiber (MMF, FG200LCC, Thorlabs, USA) attached to an XY scanning stage (CXY1, Thorlabs, USA). The MMF was guided through the central hole of a custom-made spherically-focused detector based on PVDF-TRFE foil (foil thickness of 20 µm, detector focal distance of 7 mm, aperture radius of 3.2 mm, hole diameter of 1.2 mm). Two perpendicularly arranged stages (y direction: DDSM50-M, Thorlabs, USA; x direction: M683, Physikintrumente, Germany) scanned the transducer and the fiber tip over the imaged FOV. A data acquisition card (M4i4420-X8, Spectrum, Germany) recorded both US and PD signals at a sampling frequency of 250 MHz. A custom written MATLAB 2020b code running on a PC (Intel NUC, 64 Gb RAM, Intel Core i7–10710 U) controlled the data acquisition.

For imaging and calibration, the fast-moving stage (x direction) oscillated between the boundaries of the defined FOV while the slow-moving stage incrementally moved after each B scan. A microcontroller (Teensy 3.6, PCJR, USA) monitored the position of the fast-moving stage and generated a position trigger signal after each incremental movement (Fig. 1a). A second microcontroller generated a burst mode pulse following each position trigger, which allowed acquisition of the generated optoacoustic waveforms at all three wavelengths in a single overfly scan. The individual lasers fired with a short temporal delay to avoid crosstalk between US waves emitted at different wavelengths while keeping the lateral transducer displacement between the acquisition of different wavelengths below 1 µm [10].

Per-pulse-energy (PPE) values and timepoints of laser emission were extracted from the measurements performed with the PD. The detected waveforms were corrected for both energy and time fluctuations on a pulse-to-pulse basis. Bandpass filtering was performed between 1 MHz and 90 MHz to reduce noise in the acquired bipolar waveforms.

### 2.2 Calibrated TIR

For the calibration measurement, an individual microsphere (10 µm – 20 µm, BKPMS-1.2, Cospheric, USA) was carefully picked with the tip of a needle and positioned on top of ultra-clean 1% agarose (A4718, Sigma-Aldrich, Germany) in a Petri dish (Fig. 1b). To fix the position of the sphere for the measurement, a small droplet of agar was added on top. Deionized water at room temperature was then added as an acoustic coupling medium and the Petri dish was positioned in a vertical motorized stage (MTS50M-Z8, Thorlabs, USA). For the calibration experiment, the center position of the microsphere was located along the acoustic axis before calibration experiment (Fig. 1c). During an automatic calibration procedure, the vertical stage moved the microsphere to different axial positions relative to the focal plane with a step size of 20 µm (Δz in Fig. 1c). For each depth, the transducer was scanned laterally over a 1 mm^2^ area with a bilateral step size of 20 µm (Δx in Fig. 1c). At each lateral scan position, the signals generated by the sub-resolution sized microsphere were collected and averaged 3 times to improve SNR. This procedure resulted in the acquisition of a four-dimensional cTIR. Note that scanning the transducer while keeping the microsphere fixed is equivalent to moving the microsphere to different positions relative to a stationary transducer. An isotropic step size of 20 µm ensured that the spatial discretization matched the full frequency bandwidth of the transducer. The 532 nm laser operating at a PPE of ∼100 μJ illuminated the sphere from the side to eliminate potential reconstruction artifacts arising from non-stationary illumination (Fig. 1b). The calibration experiment lasted for about 30 min in total.

### 2.3 Simulated TIR

**Fig. 2:**
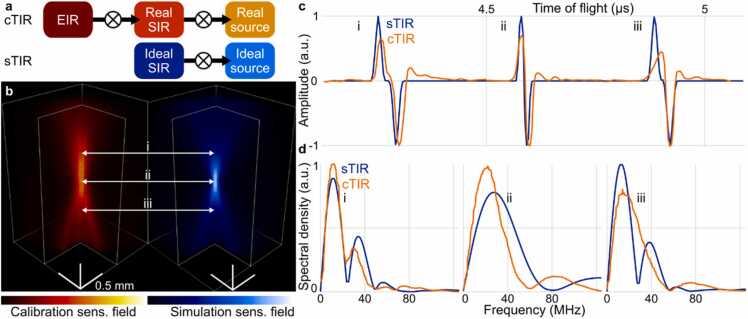
Comparison of the calibrated and simulated TIR. a) The simulation corresponds to the convolution of an ideal SIR with an ideal (theoretical) optoacoustic point source. cTIR accounts for the real EIR and SIR of the transducer and the real signal generation effects. b) Rendering of the transducer sensitivity field for the cTIR and sTIR, calculated as the maximum amplitude projections (MAPs) along the time axis. c) Measured pressure time series along the acoustic axis of the transducer at three different vertical positions along the acoustic axis. d) Frequency spectra of the waveforms shown in panel c. The frequency response of the transducer can be found in panel d-ii, where the sphere is at the focus position.

The optoacoustic signals emitted by a point source and sensed by the US detector were simulated for different field positions ${\vec{p}}_{f}$ as described in [27]. For this, the spherical detector surface ${\vec{p}}_{s}$ was divided into $n_{e}$ virtual elements with defined center positions. Specifically, the polar angle was divided equally into 25 sub-angles and the azimuthal angle was divided equally into 40 sub-angles, resulting in a total of 1000 virtual elements. Based on the transducer size specified above, individual virtual elements have an estimated average size of 50 µm^2^. The distance to all the virtual elements was calculated for each field point ${\vec{p}}_{f}$. The SIR is then defined as the histogram of the distances weighted by the element size. The time pressure series defining the sTIR were then calculated by convolving the estimated SIR with the theoretical optoacoustic waveform detected from a point absorber (Fig. 2a). To capture the laterally-confined sensitivity field of the transducer while maintaining the memory efficiency of the sTIR algorithm, the simulation was performed for voxel positions closely centered around the acoustic focus (1 mm maximum lateral radius, 3 mm axial distance). Reconstruction of a larger lateral FOV is enabled by capitalizing on the translational symmetry in the relative positions between the transducer and voxel grid [27]. The frequency responses of the sensing element and amplification system were assumed to be constant over the full frequency range. Frequency-dependent attenuation and dispersion of US waves were not considered in the simulations.

### 2.4 Reconstruction procedure

The theoretical foundation of the MBR framework was previously described elsewhere [27]. For all reconstructions shown in this work, the L2 norm of reconstructed image was used as the regularization term in the iterative inversion algorithm. Iterative inversion was performed with the LSQR procedure [28]. Both sMBR and cMBR were executed with 20 iterations of the LSQR algorithm. The number of iterations was chosen as a tradeoff between residual minimization (an indicator of image quality) and computation time. The residual norm reached convergence after 20 iterations for both sMBR and cMBR approaches (Fig. S1). The discretization grid size and scanning step size of 20 µm in lateral and axial directions were used for both cTIR- and sTIR-based reconstruction approaches. Temporal sampling frequency of 250 MHz was used for acquiring the raw data and building the transducer response in all reconstructions.

The two computationally intensive calculations were customized by converting the estimated absorbance distribution into a signal matrix and the corresponding transpose operation. The mathematical operations performed along the x-y spatial directions effectively break down into a convolution with a fixed kernel for each time-depth pair. Since the same kernel can be used for each of those slices, shared memory access has allowed to significantly reduce global memory access hence accelerating the reconstructions. With the above computational procedure, the reconstruction could be performed on a GPU (implementation through CUDA 11.0, calculation on RTX3090, NVIDIA, USA) within 20 min for a typical imaging volume spanning 10 mm by 10 mm in the lateral directions at 20 µm bilateral resolution. The code used for the acoustic inversion was written in C+ + and CUDA and is available online including a graphical user interface and data preview (https://github.com/hofmannu/openmbr).

### 2.5 Phantom scans

We measured a microsphere phantom to quantitatively assess performance of the proposed reconstruction paradigm. To avoid the inverse crime, a microsphere of a larger size (27 µm – 35 µm, BKPMS-1.2, Cospheric, USA) was scanned as described for the TIR calibration measurements. While the calibration was performed using static illumination from the side (Fig. 1b), the phantom was illuminated by the MMF guided through the central hole with the acoustic axis and illumination cone co-aligned during the scan. To account for deviations from the rotational symmetries of the TIR around the acoustic axis, the transducer was kept in the same orientation during calibration and phantom measurements. The time-resolved signals corresponding to different axial positions of the sphere were summed up. The microsphere was scanned in a region of 1.5 mm by 1.5 mm in x and y, respectively, at a bilateral step size of 20 µm. The 532 nm laser operating at a PPE of approximately 100 μJ was used as excitation source. No averaging was performed. Each scan for a particular position of the sphere took approximately 20 s

### 2.6 Mouse and human skin scans

Imaging was performed on the back of a nude mouse for a FOV spanning a 30 mm by 12 mm area at a bilateral step size of 20 µm. The laser source at 532 nm operating at a PPE of 25 μJ was used at a peak pulse repetition frequency (PRF) of 5 kHz. The same region was imaged with optical-resolution optoacoustic microscopy setup [29] prior to performing the OAM scans. All animal experiments were performed in accordance with the Swiss Federal Act on Animal Protection and were approved by the Cantonal Veterinary Office Zurich.

The human skin was imaged at the forearm of a healthy volunteer (Fitzpatrick scale type II) for a FOV spanning across 20 mm by 10 mm at a bilateral step size of 20 µm. The volunteer was informed and gave written consent. The laser source at 532 nm operating at a PPE of 25 μJ was used at a peak PRF reaching 2.5 kHz, thus maintaining the laser illumination levels below the safety limits for human skin exposure [30]. For better visualization of the vascular tree, a Frangi filter was applied to the reconstructed datasets.

## 3 Results

### 3.1 Comparison between calibrated and simulated TIR

The experimental calibration procedure effectively accounts for all the effects involved in the optoacoustic signal excitation, the subsequent acoustic propagation and detection (Fig. 2a). This includes properties of the laser source as well as the EIR and the SIR of the transducer. In contrast, the sTIR is based on the geometrical calculation of the SIR assuming an ideal source (Dirac’s delta excitation). Small deviations from these assumptions may lead to noticeable differences between the sensitivity fields (Fig. 2b). The sensitivity field of the cTIR revealed asymmetries in lateral and axial directions around the acoustic axis. These were not correctly reflected by the simulations assuming cylindrical symmetry.

A comparison between simulated (theoretical) and measured (calibrated) signals generated by the sphere located along the acoustic axis further underscores the discrepancies between the sTIR and cTIR approaches (Fig. 2c). The microsphere signal measured in the focal point - herein termed focal signal - correlated well with the modelled N shape signal, both in time and frequency domains (Fig. 2c ii). The disparity may arise from manufacturing imperfections which contributed to non-uniformity of the detector’s sensitivity field. Part of the problem may have arisen due to asymmetric stretching of a flat PVDF foil to form an approximate spherical shape. Furthermore, the sTIR does not account for spatially- and frequency-dependent attenuation and dispersion of US waves, both of which were captured by the calibration measurement. The calibration further shows asymmetries across the far and near fields that cannot be modelled through the frequency dependent EIR or US attenuation.

### 3.2 cMBR improves depth uniformity, SNR, and lateral resolution

**Fig. 3:**
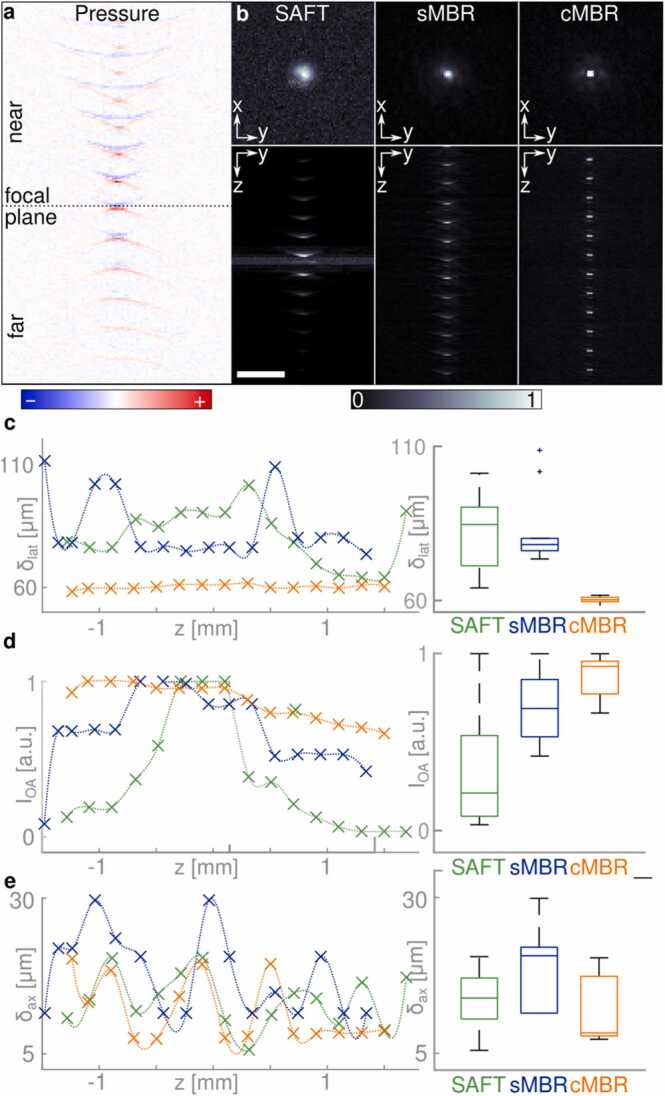
Performance of the developed reconstruction approach in phantom measurements. a) Individual microspheres measured at different vertical positions. The measured signals were added up. b) Maximum amplitude projections (MAPs) of the datasets reconstructed with SAFT, sMBR, and cMBR (scale bar: 250 µm). c) Lateral resolution versus depth. The resolution is defined as the full width at half maximum (FWHM) of the reconstructed spheres. d) Reconstructed intensity versus depth. e) Axial resolution versus depth.

cMBR performance was compared against SAFT and sMBR in phantom measurements. For this, a target containing a 25 µm diameter microsphere embedded in 1% ultra-pure agarose was scanned with the detector aligned in the same orientation as in the calibration measurement. The microsphere signals corresponding to different axial positions were summed up to simulate multiple microspheres positioned along the depth direction (Fig. 3a). The measured time domain signals were converted into volumetric reconstructions by means of the SAFT, sMBR, and cMBR algorithms. The MBR methods were executed by running through 20 iterations of the LSQR procedure.

Owing to asymmetries of the detector sensitivity field, the images reconstructed with the sMBR method feature directional, side-lobe artifacts around the spheres (Fig. 3b). Since cMBR automatically incorporates these inaccuracies, no such artefacts were observed in the corresponding reconstructions.

The lateral and axial resolutions at different axial locations were calculated to assess performance of the different reconstruction approaches (Fig. 3c). SAFT achieved an average lateral resolution of (80.8 µm ± 11.1 µm), which was outperformed by both the sMBR (75.2 µm ± 9.8 µm) and cMBR (60.3 µm ± 0.9 µm). Hence, significant improvement in lateral resolution was achieved by both sMBR and cMBR (two-sided student’s t-test, p < 0.001) with the resolution also being highly uniform over a large depth range spanning approximately 3 mm around the focal plane.

The so-called depth uniformity, namely, the OAM image intensity versus depth, was numerically evaluated as the difference between the highest and lowest signal recovered from the sphere positioned at different axial locations (Fig. 3d). cMBR allows for a quantitative comparison between features located at various distances from the focal plane. The microspheres reconstructed with SAFT show the largest span of the reconstructed initial pressure distribution reaching 97% intensity drop as the microsphere moves away from the focal point. The maximum intensity reduction is 58% and 33% for sMBR and cMBR respectively, highlighting the improved depth uniformity achieved with cMBR. The remaining intensity decay with depth is ascribed to the different illumination schemes used for the static calibration and the scanning phantom measurements - in the latter case, spheres at deeper layers are positioned further away from the fiber tip resulting in reduced optical fluence.

It should be noted that the optimal number of neighboring A scans required for the SAFT reconstruction depends on the distance from the focal plane. This results in an increased noise background around the focal plane where an overlap between neighbouring scans is minimal. The iterative inversion approaches enabled an improved and, more importantly, uniform SNR over the full depth range.

### 3.3 cMBR improves mouse skin angiography

**Fig. 4:**
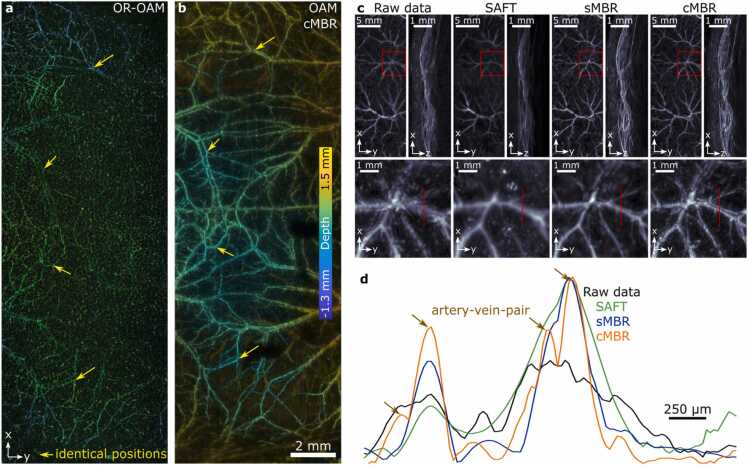
Comparison of different reconstruction methods for OAM-based angiography of the mouse skin. An identical FOV was imaged with a) optical-resolution optoacoustic microscopy (OR-OAM) and b) optoacoustic mesoscopy (OAM) using cMBR. The yellow arrows indicated superficial vasculature which could be identified with both approaches. Since OAM does not employ focused beams, it is not directly affected by the intense light scattering in the skin thus can resolve vasculature far beyond the limits imposed by optical diffusion. c) Raw data and performance of different reconstruction approaches. Data is shown as maximal amplitude projection (MAP) images. d) Cross-section through an artery-vein-pair: The resolution improvement through cMBR allows rendering two separate vessels, while all other approaches erroneously merge them into a single vessel.

The benefits of cMBR were subsequently demonstrated by OAM imaging of a 30 mm x 12 mm FOV on the mouse dorsal skin at a bilateral step size of 20 µm without signal averaging. The same area was imaged with an OR-OAM system [29]. While superficial structures can be resolved with high spatial resolutions by OR-OAM, deeper structures remain invisible due to the rapid loss of signal resulting from broadening of the narrowly focused optical beam (Fig. 4a). In contrast, more extensive and deeper vascular network is visible with OAM as a result of more uniform illumination of deeper tissue layers (Fig. 4b).

When comparing the different reconstruction approaches (Fig. 4c), structures positioned in the focal plane are readily resolved with high spatial resolution when presenting the raw data as simple maximal amplitude projections. However, smaller structures away from the focal plane get blurred and presented with diminished intensity. The SAFT approach improves the SNR while mitigating background noise of the image. Nevertheless, the images are afflicted with the lowpass filtering effect of SAFT. The sMBR approach improved the image quality beyond SAFT and accurately reconstructed smaller vascular structures. The sMBR approach allowed a clear and accurate representation of the full vascular network at different z layers (depths). Both sMBR and cMBR clearly recover signals from deeper vessels, which cannot be identified with SAFT.

When inspecting one-dimensional image profile through an artery-vein pair (Fig. 4d), the two vessels can only be resolved with cMBR whereas the low pass filtering effect of SAFT and characteristic side-lobe artifacts of sMBR hinder the separation of the vessels.

The broadband sensitivity of the PVDF detector enables an accurate recording of ultrawideband optoacoustic signals generated by vessels of different sizes. Since SAFT acts like a lowpass filter, it requires separate reconstruction over different frequency bands [31]. In contrast, the cMBR approach can handle the entire frequency spectrum within a single acoustic inversion procedure.

### 3.4 Towards quantitative acoustic inversion for human skin imaging

**Fig. 5:**
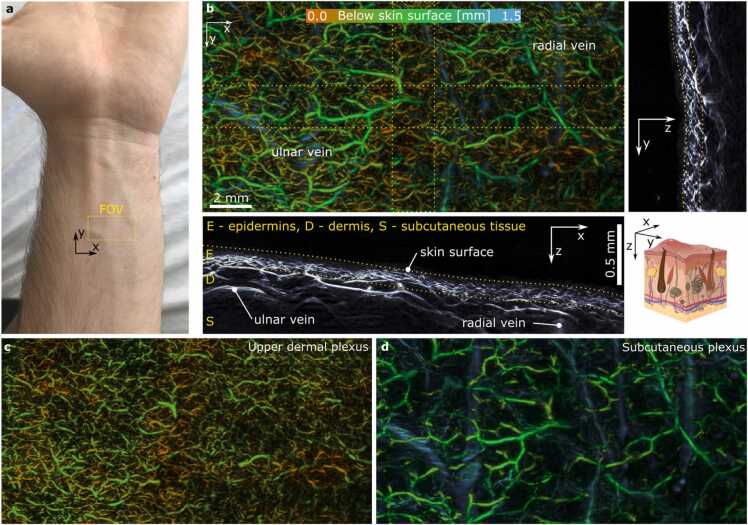
Application of cMBR to recover the vasculature in human skin. a) photograph of the forearm imaged with the system with the yellow box indicating the approximate FOV. b) Depth encoded MAP along vertical domain allows visualization of a dense vascular network. The depth was encoded starting from the skin surface which was extracted from the low frequency signal originating from the superficial melanin layer. The side views show cross sections including deep structures such as ulnar and radial vein positioned below the dermis. c) and d) Subdivision of the upper and lower vascular network based on the distance from the skin surface.

The deep penetration of an unfocused excitation light makes OAM a promising tool for human skin angiography. To showcase the clinical potential of the cMBR approach, skin of a healthy volunteer was imaged in the forearm region (Fig. 5a), resulting in a clear visualization of the dense vascular network (Fig. 5b). For analysis, we extracted the skin surface from the highly absorbing melanin layer in the epidermis and assigned a color scheme representing depth from the skin surface, which facilitated a clear separation between two distinct vascular networks. The upper dermal plexus consists of small arterioles and venules supplying the upper skin layer. The deeper subcutaneous plexus layer consists of larger vessels supplying the superficial network. By using the information of the skin depth, the two vascular networks could be segmented by applying a 0.5 mm depth segmentation (Fig. 5c and d). The radial and ulnar vein positioned approximately 2 mm below the surface could be partially reconstructed from the acquired raw datasets, thus clearly indicating the potential of the technique to recover vasculature over the full thickness of human skin.

## 4 Discussion

We presented a new reconstruction paradigm for OAM based on a calibration measurement used during the iterative acoustic inversion procedure. The devised approach overcomes limitations imposed by inaccurate modeling of US transducer response, further accounting for the frequency-dependent EIR of the amplification electronics as well as the properties of the laser excitation patterns. Beyond the observed improvements in the lateral and axial resolution, the cMBR-based reconstruction represents an important step towards quantitative OAM imaging and spectroscopy by accurately inverting the acoustic problem across broad depth ranges. By making use of GPU parallelization, iterative inversion of large volumetric datasets can be performed in a matter of minutes, allowing a practical use of the reconstruction procedures both in preclinical research and time-critical clinical settings.

Reconstructions made from calibration phantom measurements revealed that the most commonly applied SAFT-based algorithms have resulted in major intensity shifts over depth, thus hindering quantitative interpretation of the OAM results. Without correction for those reconstruction artifacts, the acquired images are prone to misinterpretation whereas imaging of objects that are not perfectly aligned with respect to the focal plane would necessarily lead to arbitrary results, both from a quantitative and qualitative points of view.

The calibrated TIR presented in this work incorporates the acoustic propagation characteristics of the calibration phantom. The effective acoustic properties may differ for various samples. In particular, biological tissues exhibit stronger acoustic attenuation at high frequencies as compared to the agarose medium used in the calibration experiment. Furthermore, the speed of sound heterogeneities result in temporal shifts of the recorded signals thus location shifts of the reconstructed absorbers.

The depth of the reconstructed spheres differs between cMBR and sMBR (see Fig. 3a). For the simulation studies, the transducer is modeled with a perfect spherical shape and the absorber is positioned accurately at different lateral and axial positions around the focal point. While measuring the cTIR, relative distances are determined based on the stage positions but absolute vertical and lateral distances between transducer and sphere remain unknown. Depth shifts observed in the reconstruction of the phantom therefore result from inaccuracies in determining the focal plane of the used sensor from the cTIR dataset.

While the presented methods accurately account for the acoustic inversion part of the OAM problem, the optical propagation problem has not been considered as part of the MBR inverse procedure. Future work will thus expand the demonstrated methodology by inverting the combined optical and acoustic problem in a single coupled procedure, e.g. by accounting for the moving illumination source in the mesoscopic light propagation range by means of fast Monte Carlo simulations [32], [33]. Through this, additional reconstruction inaccuracies, such as those resulting from spectral coloring effects [34], [35], can be further mitigated.

The developed paradigm of inverting the acoustic problem through a calibration measurement can be expanded to more sophisticated US detectors [36], [37], to other microscopic and tomographic optoacoustic imaging modalities [4], [38], and, potentially, additional imaging methodologies relying on iterative inversion schemes. In particular, US detectors having solid lenses where longitudinal–shear wave conversion often occur are particularly hard to model. cMBR offers a practical option to boost the reconstruction performance of data obtained from such transducers.

In summary, the presented work demonstrated the feasibility of using a simple microsphere-based calibration measurement to retrieve comprehensive spatio-temporal characteristics of the imaging system thus facilitating improved reconstruction performance and averting the need for complex ill-posed physics-based simulations.

## CRediT authorship contribution statement

**Urs A.T. Hofmann:** Conceptualization, Visualization, Software, Data curation, Methodology, Writing - original draft, Writing - review & editing. **Weiye Li:** Software, Methodology, Visualization, Writing - original draft, Writing - review & editing. **Xosé Luís Deán-Ben:** Software, Funding acquisition, Methodology, Writing - original draft, Writing - review & editing. **Pavel Subochev:** Funding acquisition, Writing - review & editing. **Héctor Estrada:** Conceptualization, Supervision, Visualization, Writing - original draft, Writing - review & editing. **Daniel Razansky:** Supervision, Funding acquisition, Writing - original draft, Writing - review & editing.

## Declaration of Competing Interest

The authors declare that they have no known competing financial interests or personal relationships that could have appeared to influence the work reported in this paper.

## Data Availability

Data will be made available on request.
