# Supplementary material for: Enhancing optoacoustic mesoscopy through calibration-based iterative reconstruction
Source: Photoacoustics. 2022 Sep 23;28:100405. doi: 10.1016/j.pacs.2022.100405 (PMC9554813; doi:10.1016/j.pacs.2022.100405)
Supplement: Supplementary file 1 — Supplementary material [file mmc1.docx]

Supplementary Figure

**Enhancing optoacoustic mesoscopy through calibration-based iterative reconstruction**

Urs A. T. Hofmann, Weiye Li, Xosé Luís Deán-Ben, Pavel Subochev, Héctor Estrada, and Daniel Razansky


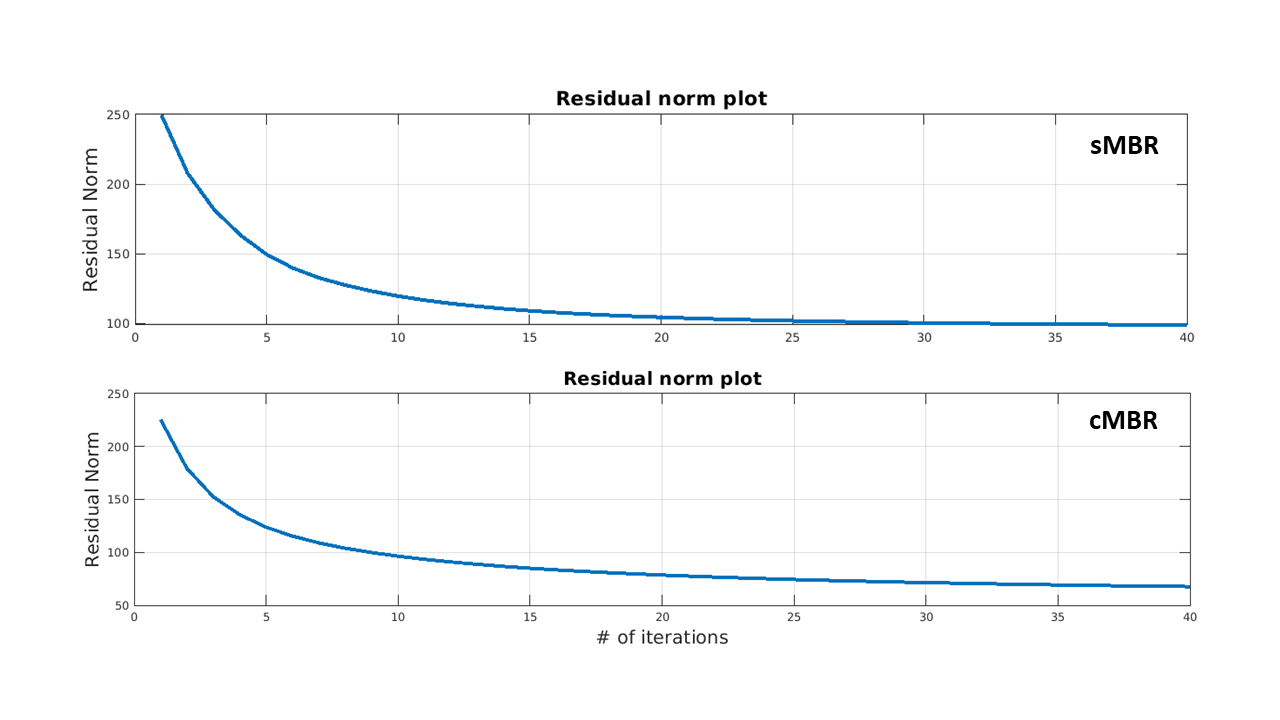


Figure S1: Convergence analysis of both sMBR and cMBR executed over 40 iterations of the LSQR algorithm on a representative mouse dorsal skin dataset. The evolution of the residual norm is plotted as a function of the number of iterations. The images shown in the manuscript were reconstructed after 20 iterations for both sMBR and cMBR, representing a good trade-off between residual norm minimization (an indicator of image quality) and computation time.
